# The importance of considering cultural and environmental elements in an interventional model of care to fight hypertension in Africa

**DOI:** 10.1111/jch.14252

**Published:** 2021-04-08

**Authors:** Pauline Cavagna, Kouadio Eulodge Kramoh, Abdallahi Sidy Ali, Dahdi M. Balde, Abdoulaye K. Traore, Stephanie Khoury, Xavier Jouven, Marie Antignac

**Affiliations:** ^1^ Department of Pharmacy St Antoine Hospital AP‐HP Sorbonne Université Paris France; ^2^ INSERM U970 Paris Cardiovascular Research Center Université de Paris Paris France; ^3^ Institute of Cardiology of Abidjan Abidjan Côte d'Ivoire; ^4^ Centre National de Cardiologie Cabinet de Cardiologie Nouakchott Mauritania; ^5^ Cardiology Department University Hospital of Conakry Conakry Guinea; ^6^ Cardiology Department Hospital of Sikasso Sikasso Mali; ^7^ Cardiovascular Epidemiology Department Université de Paris Paris France; ^8^ Cardiology Department AP‐HP Centre European Georges Pompidou Hospital Paris France

To the Editor,

We read with great interest the article by Otieno et al,^1^ titled “Improved blood pressure control via a novel chronic disease management model of care in Sub‐Saharan Africa: Real‐world program implementation results” which presents a novel hypertension management model of care to improve blood pressure (BP) control in sub‐Saharan Africa. This model of care is a real innovation that uses smartphones and regular check‐ins to ensure optimal patient follow‐up. This study, implemented in two middle‐income countries, successfully improved and sustained BP control over 12 months of follow‐up.

Firstly, this model of care, by standardizing the management of hypertension, placed patients at the heart of their care. This model of care developed a direct link between the patient, the physician, and the pharmacy. However, it did not include a traditional health practitioner and it did not seem to take into account the use of traditional medicine. The World Health Organization defines the use of traditional medicine as the sum total of the knowledge, skill, and practices based on the theories, beliefs, and experiences indigenous to different cultures, whether explicable or not, used in the maintenance of health as well as in the prevention, diagnosis, improvement, or treatment of physical and mental illness.^2^ The use of traditional medicine is documented in both African countries and worldwide with a prevalence varying between 20% and 80%.^3^, ^4^, ^5^, ^6^, ^7^ Among African hypertensive patients, the use of traditional medicine is common without an association with age or educational levels.^5^ In a recent multinational study on sub‐Saharan Africa hypertensive patients, the use of traditional medicine was shown to be strongly associated with poor adherence to conventional treatments.^8^ It would be an interesting and useful addition to include traditional medicine and traditional health practitioners in the model of care presented by Otieno and colleagues. Although this model of care has greatly improved hypertension management in sub‐Saharan Africa, it can be improved further by considering other cultural and environmental aspects of Africa, particularly the use of traditional medicine and health practitioners.^9^

Secondly, in low‐ and middle‐income countries, access to medication is defined by five dimensions (availability, affordability, accessibility, acceptability, and quality of drugs^10^). In this setting, a large proportion of communities do not have access to more than one antihypertensive drug and, when they are available, they are often not affordable.^11^ In sub‐Saharan Africa, access to medicine is an especially crucial element in a model of care performed to improve BP control, and in this intervention, access to medication is absolutely deleted.

Finally, this model of care was implemented across rural and urban facilities in two middle‐income countries, whereas African countries are largely classified as low income. It is worth considering whether this model could be generalized to low‐income countries as the wealth index of patients has been associated with different levels of hypertension control.^12^

## CONFLICTS OF INTEREST

No author has any competing interests.

## AUTHOR CONTRIBUTIONS

All authors have substantial contributions.

P. Cavagna, M. Antignac, S. Khoury, and X. Jouven drafted the manuscript. All authors critically revised the manuscript for important intellectual content and approved the final version to be published.
